# Can mental health treatments help prevent or reduce intimate partner violence in low- and middle-income countries? A systematic review

**DOI:** 10.1186/s12905-019-0728-z

**Published:** 2019-02-14

**Authors:** W. A. Tol, S. M. Murray, C. Lund, P. Bolton, L. K. Murray, T. Davies, J. Haushofer, K. Orkin, M. Witte, L. Salama, V. Patel, G. Thornicroft, J. K. Bass

**Affiliations:** 1 Department of Mental Health, Johns Hopkins Bloomberg School of Public Health, 624 N Broadway, HH795, Baltimore, MD 21205 USA; 2 Peter C. Alderman Program for Global Mental Health, HealthRight International, New York, USA; 3 Alan J Flisher Centre for Public Mental Health, Department of Psychiatry and Mental Health, University of Cape Town, Cape Town, South Africa; 4 Centre for Global Mental Health, Health Service and Population Research Department, Institute of Psychiatry, Psychology and Neuroscience, King’s College London, London, UK; 5 Department of International Health, Johns Hopkins Bloomberg School of Public Health, Baltimore, USA; 6 Department of Psychology and Woodrow Wilson School of Public and International Affairs, Princeton University, Princeton, NJ USA; 7 National Bureau of Economic Research, Cambridge, MA USA; 8 Busara Center for Behavioral Economics, Nairobi, Kenya; 9 Blavatnik School of Government and Centre for the Study of African Economies, University of Oxford, Oxford, UK; 10 Merton College, University of Oxford, Oxford, UK; 11 Department of Economics and Centre for the Study of African Economies, University of Oxford, Oxford, UK; 12 Department of Global Health and Social Medicine, Harvard Medical School, Boston, USA; 13 Department of Global Health and Population, Harvard Chan School of Public Health, Boston, USA

**Keywords:** Mental health, Intimate partner violence, Low- and middle-income countries, Treatment, Multisectoral interventions, Systematic review

## Abstract

**Background:**

Epidemiological research suggests an interrelationship between mental health problems and the (re)occurrence of intimate partner violence (IPV). However, little is known about the impact of mental health treatments on IPV victimization or perpetration, especially in low- and middle-income countries (LMIC).

**Methods:**

We conducted a systematic review to identify prospective, controlled studies of mental health treatments in LMIC. We defined ‘mental health treatment’ as an intervention for individuals experiencing mental ill health (including substance misuse) including a substantial psychosocial or pharmacological component. Studies had to measure a mental health and IPV outcome. We searched across multi-disciplinary databases using a structured search strategy. Screening of title/abstracts and full-text eligibility assessments were conducted by two researchers independently, data were extracted using a piloted spreadsheet, and a narrative synthesis was generated.

**Results:**

We identified seven studies reported in 11 papers conducted in five middle-income countries. With the exception of blinding, studies overall showed acceptable levels of risk of bias. Four of the seven studies focused on dedicated mental health treatments in various populations, including: common mental disorders in earthquake survivors; depression in primary care; alcohol misuse in men; and alcohol misuse in female adult sex workers. The dedicated mental health treatments targeting depression or alcohol misuse consistently reduced levels of these outcomes. The two studies targeting depression also reduced short-term IPV, but no IPV benefits were identified in the two alcohol-focused studies. The other three studies evaluated integrated interventions, in which a focus on substance misuse was part of efforts to reduce HIV/AIDS and violence against particularly vulnerable women. In contrast to the dedicated mental health interventions, the integrated interventions did not consistently reduce mental ill health or alcohol misuse compared to control conditions.

**Conclusions:**

Too few studies have been conducted to judge whether mental health treatments may provide a beneficial strategy to prevent or reduce IPV in LMIC. Key future research questions include: whether promising initial evidence on the effects of depression interventions on reducing IPV hold more broadly, the required intensity of mental health components in integrated interventions, and the identification of mechanisms of IPV that are amenable to mental health intervention.

**Electronic supplementary material:**

The online version of this article (10.1186/s12905-019-0728-z) contains supplementary material, which is available to authorized users.

## Background

Intimate partner violence (IPV) is a critical human rights and public health concern. IPV refers to behavior within an intimate relationship that causes, or has the potential to cause, physical, sexual, or psychological harm, including acts of physical aggression, sexual coercion, psychological abuse, and controlling behaviors [1]. A comprehensive meta-analysis of 141 studies from 81 countries found that 30% of women and girls aged 15 and older have experienced IPV [2]. Consequences of IPV include physical, reproductive, and mental health issues [3–5] and in severe cases, the resulting injuries can be fatal [6]. Knowledge is accruing on how to best prevent and reduce IPV [7]. Research from low- and middle-income countries (LMIC) has more frequently focused on preventive interventions, and has shown promising benefits of group training for men and women (e.g., participatory learning activities focused on gender roles and conflict resolution skills), community mobilization interventions, and combined livelihood and training interventions for women [8]. With regard to efforts to reduce IPV once detected, evidence (mainly from high-income countries) suggests that women-centered care, advocacy, and home-visitation programs can reduce the risk of further victimization [8, 9]. Although treatment of mental ill health or substance abuse may strengthen efforts to prevent and reduce IPV [10] relatively little research has focused on this topic.

There are several reasons to think that treatment of mental disorders and substance abuse problems may be an effective strategy for prevention and reduction of IPV in LMIC either through targeting perpetrators or victims. Hazardous alcohol use [11–14], common mental disorders (posttraumatic stress disorder [PTSD], depression, anxiety) [12–16], and anger dysregulation [17] are known correlates of IPV perpetration. Yet, attention to perpetrators’ mental disorders has not commonly been included in batterer intervention programs such as the Duluth model. Duluth interventions tend to focus on gender reeducation aimed at addressing the patriarchal factors underlying male perpetration of IPV. Evaluations of traditional batterer intervention programs based on this model, commonly in high-income countries, have shown conflicting results [18]. Interventions that include components to address perpetrators’ mental ill health may strengthen the effectiveness of efforts to stop or reduce IPV, given the strong correlations of mental health with IPV perpetration [19–22].

Mental health interventions may also reduce further risk for victimization by treating mental health problems among IPV survivors [23–25]. Longitudinal studies suggest that the relationship between IPV and mental ill health may in fact be cyclical: mental health impacts of IPV put women at increased risk for further abuse [26–29]. For example, depression may be associated with self-blame for IPV victimization, reduced self-esteem, and hopelessness. Similarly, PTSD symptoms such as emotional numbing may challenge survivors’ ability to detect or respond to IPV risks [28, 30]. Mental health interventions therefore may reduce IPV re-victimization by targeting mental ill health in survivors [21]. Consistent with this hypothesis, a randomized controlled trial from the United States, providing cognitive behavioral therapy to interpersonal violence survivors reduced IPV re-victimization [30]. Both for survivors and perpetrators, mental health treatments may have additional indirect benefits for IPV reduction by conferring psychological and social skills - strengthening communication, stress management, and anger management skills, and reducing social isolation – that may reduce IPV incidence [31].

We follow a multidimensional (“both/and”) perspective, where attention to mental health occurs as part of an analysis of the various factors – both individual and structural -- that contribute to IPV [22]. To date, less attention has been placed on some of the individual-level factors (e.g., witnessing domestic violence in childhood, experiencing child abuse, alcohol abuse) which are strongly and consistently related to IPV [12, 13, 21, 32]. We note that interventions addressing the mental health of survivors need to be mindful of the risk of victim blaming, i.e. pointing to individual characteristics associated with higher risk for IPV without acknowledging the broader structural forces that confer risk for IPV. We also highlight the wider constellation of risk and protective factors for IPV [33] noting both family- and community-level factors (e.g., relationship practices, household poverty) [12, 21] and broader socio-cultural factors (e.g., gender-inequitable social norms and traditional notions of masculinity) [12, 13, 34] which are important correlates of IPV perpetration and victimization. We examine individual-level factors, in particular mental health, without diminishing the importance of these broader social and structural influences.

Research findings from high-income countries may not generalize to LMIC because of differences in the distribution of determinants of violence; socio-cultural context; resources available to respond to IPV; notions of mental illness; and characteristics of mental health systems. Given the potential of mental health interventions to address IPV and the gap in knowledge on this topic in LMIC, in this systematic review we synthesize findings from controlled trials of mental health interventions conducted in LMIC that included IPV as a primary or secondary outcome.

## Methods

### Inclusion and exclusion criteria

The protocol for this systematic review was registered with PROSPERO (2017: CRD42017064660). We included prospective, controlled studies (either via comparison group or statistical design that enabled “self control” comparisons) that assessed the impact of mental health treatments on IPV among a sample primarily composed of adolescents and adults (at least 50% of the sample was age 10 or older) in LMIC. Low and middle-income countries were defined using the latest World Bank income classifications, including both lower- and upper-middle income countries. An intervention was considered a mental health treatment if it met all of the following criteria: 1) included a mental health component, i.e. an element theorized explicitly by study authors to target mental health symptoms or substance use; 2) evaluated a pharmacological or psychosocial program delivered to individuals screened in on the basis of mental ill health or substance misuse, either by using a defined disorder diagnosis or scoring above a pre-defined cutoff on a screener for symptoms of disorder or general psychological distress; 3) measured a mental or substance use disorder or symptom as an outcome; and 4) included a measure of IPV, either physical, sexual or psychological, as a study outcome (primary or secondary). We excluded studies where violence was measured as occurring between: people in the general community; paying sexual partners; or family members that were not intimate partners (i.e. father and child, mother and daughter-in-law, elder abuse within families). We did not set any restrictions by year of publication.

Trials with all types of inactive control conditions were considered for inclusion, including placebo, waitlist, no treatment, treatment as usual, or treatment without an active mental health component. We also included studies with only one treatment arm that adequately controlled for unobserved confounding in design and analysis (e.g. regression discontinuity designs, instrumental variable approaches, difference-in-difference designs, or interrupted time series). We excluded studies that compared two or more active treatments without a control condition. We excluded non-peer reviewed literature (e.g., book chapters and dissertations). We excluded studies that did not have an abstract in English. If an article had an abstract in English but was written in any other language, the article was still eligible for inclusion.

### Search strategy

Our search strategy combined terms aimed at identifying studies that: (1) were conducted in LMIC; *and* (2) evaluated mental health treatments (i.e. had a mental health or psychosocial component and were delivered to individuals experiencing ill mental health); *and* (3) assessed IPV as an outcome; *and* (4) were controlled prospective studies. To ensure identification of studies conducted in LMIC, we applied a set of keywords developed by Johns Hopkins University librarians to include general terms used to describe LMIC (e.g. developing country, less developed nation, third world) as well as the names of all countries classified by the World Bank as low- or middle-income.

To identify studies focused on mental health treatments, search terms included names of mental disorders, categories of disorders, and commonly abused substances, as well as general terms for mental ill health (e.g. psychological stress, aggression, mental disorders). To ensure identification of studies assessing IPV outcomes, we included various terms for IPV (e.g. partner abuse, marital abuse, rape), as well as search terms describing specific forms of abuse (psychological abuse) and the broader term violence. To identify studies with intended research design, we used Cochrane recommended search terms for randomized controlled trials (see http://work.cochrane.org/pubmed) and added terms for non-randomized controlled studies and rigorously designed prospective observational studies that adequately controlled for confounding.

A search strategy was initially developed by selecting multiple medical subject headings (MeSH) terms and subheadings relevant to mental health problems (e.g. mental disorders; stress; psychological) and interventions (e.g. psychotherapy; psychotropic drug) in PubMed. Together with a university librarian, this search was iteratively refined by examining search strategies from relevant reviews (e.g. [35–37] of the impact of mental health interventions and a search of keywords of relevant and retrieved irrelevant articles. This search strategy was then adapted for use across different databases, given databases’ different restrictions on searches (e.g., limited number of terms to search) and thesauruses (e.g., the use of MeSH in PubMed). As an example, our initial PubMed/Medline search strategy is provided in Additional file 1.

Other databases searched were Web of Science (including the Social Science Citation Index); Scopus (including Medline and Embase); Ebscohost (AfricaWide, psychINFO, CINAHL); and ProQuest (PILOTS and IBSS). In addition, we hand-searched the following regional databases, trial, and funding registries: Cochrane Central Register of Controlled Trials, ClinicalTrials.gov, EU Clinical Trials Register, ISRCTN Registry, National Institute of Health (NIH) Reporter, and WHO databases (Western Pacific Region Index Medicus, WHO Global Index Medicus, South East Asia Regional Office, Eastern Mediterranean Regional Office, African Index Medicus). Additional hand searching included the reference list of any relevant systematic reviews or published trial protocols found through this search process, as well as forward and backward citation checks on any article eligible for inclusion. We also reached out to all authors of included articles to ask if they, as experts in this area, knew of any articles that we had missed in our search process.

Search results from all databases and registries were compiled and duplicates eliminated by a single researcher using Covidence software. Two researchers then independently: (1) screened titles and abstracts, and (2) screened the full text of any article that was found to be potentially eligible. In the case of conflicting decisions on eligibility, the two reviewers (SM, LS) discussed the discrepancy and their rationale. If consensus could not be reached, a third party (WT, JB) was consulted for a final decision.

### Data extraction, risk of bias, and analysis

One author (LS) extracted information from eligible full texts into a piloted, structured Excel spreadsheet. A second author (SM) checked all the extracted information, and consulted a third reviewer (WT, JB) as necessary when her interpretation of an article varied substantially from the first reviewer or the information provided in the manuscript was unclear. The data extraction spreadsheet included entries for: *sample and population characteristics* (e.g. country, sample size, demographics, mental health condition targeted); *study design and procedures* (number and timing of assessments, statistical methodology, instruments used to measure mental ill health and IPV); *intervention information* (type of intervention, mode of delivery, duration and dose, if it included a gender transformative or violence specific component, control condition); *study findings* (intervention effects for IPV and mental ill health/ substance abuse outcomes, results of any analysis of mediators, sub-group analyses); and information related to risk of bias. Risk of bias assessments were made using the Cochrane Risk of Bias Tool by two authors independently (SM, WT). This tool included the following dimensions: selection bias (sequence generation; allocation concealment); performance bias (blinding of participants and personnel); detection bias (blinding of outcome assessment); attrition bias (incomplete outcome data); and reporting bias (selective outcome reporting).

We planned to conduct a narrative synthesis and, if a sufficient number of high-quality trials were identified with sufficient homogeneity, meta-analysis using aggregate data.

## Results

**Fig. 1:**
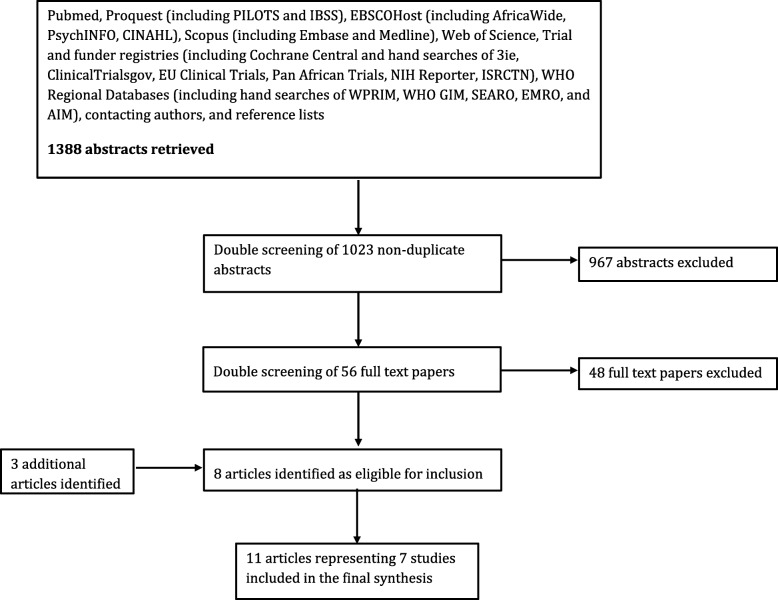
PRISMA flowchart. Pubmed, Proquest (including PILOTS and IBSS), EBSCOHost (including AfricaWide,PsychINFO, CINAHL), Scopus (including Embase and Medline), Web of Science, Trialand funder registries (including Cochrane Central and hand searches of 3ie,ClinicalTrialsgov, EU Clinical Trials, Pan African Trials, NIH Reporter, ISRCTN), WHORegional Databases (including hand searches of WPRIM, WHO GIM, SEARO, EMRO, andAIM), contacting authors, and reference lists

We screened the titles and abstracts of 1023 unique records (see Fig. 1 for the PRISMA flow diagram). Of these, we reviewed the full-texts of 56 papers for potential inclusion. Of the 56 papers, eight were determined to meet all study criteria. Three additional eligible articles were identified through cross-referencing. The final group consisted of seven studies, reported in 11 papers.

### Characteristics of included studies

An overview of the seven included studies is provided in Table 1. Studies were published between 2011 and 2017 and were conducted in five countries (two in India, two in South Africa, and one each in China, Kenya and Mongolia). Six out of the seven studies (86%) were randomized controlled trials, and one study [38] was an interrupted time series. Two of the seven studies (29%) [38, 39] were reported as pilot studies.

**Table 1:** Characteristics of included studies and interventions

Authors, year	Country	Design	Population	Sample size	Intervention	Format	Facilitators	Study arms
Dedicated mental health interventions								
Jiang et al., 2014 [39]	China	RCT	Adult men and women referred from mental health clinics or a community sample from a local high school affected by 2008 Sichuan earthquake with diagnosis of PTSD or major depressive disorder	49	Locally adapted version of interpersonal therapy (IPT). Treatment as usual (TAU): weekly medication management for any participant taking SSRI, SNRI or benzodiazepine; access to crisis counseling services	12 1-h weekly sessions delivered individually	Majority psychologist/ psychiatrist	(1) IPT and TAU; (2) TAU
L’Engle et al. 2014: [43] Parcesepe et al., 2016 [44]	Kenya	RCT	Adult female sex workers attending drop in centers identified as moderate-risk drinkers (harmful or hazardous use)	818 (565 contributed to analysis)	Adaptation of WHO Brief Intervention for Alcohol Use, informed by stages of change and social cognitive theory, including motivational interviewing and goal setting. Nutrition control condition, 6 sessions of comparable length.	6 20-min counseling sessions delivered to individuals monthly	Nurses	(1) Alcohol counseling; (2) Nutrition intervention of comparable time also delivered by nurses
Nadkarni et al. 2017a [47] and Nadkarni et al. 2017b [48]	India	RCT	Adult men who screened positive as harmful drinkers at primary health care centers	377 (301 contributed to analysis)	Counseling for alcohol problems (CAP), a manualized psychotherapy that includes teaching general and alcohol-specific cognitive and behavioral skills and problem solving with counsellor use of motivational interviewing and client-centered approach Enhanced usual care (EUC) following adapted mhGAP guidelines in which the patient and primary health care provider received alcohol screening results and referrals to specialists as necessary	Up to 4 30–45 min sessions delivered over 4–8 weeks to individuals in primary care or home as necessary	Lay counsellors with at least secondary school education	(1) CAP and EUC; (2) EUC alone
Patel et al., 2017 [45] and Weobong et al. 2017 [46]	India	RCT	Adult men and women who screened as experiencing moderately severe to severe depression at primary health care centers	495 (377 married men and women contributed to analysis)	Health Activity Program (HAP), a manualized psychotherapy based on behavioral activation. Enhanced usual care (EUC) following adapted mhGAP guidelines in which the patient and primary health care provider received screening results and made referrals to psychiatric care as necessary	6–8 30–50 min sessions delivered weekly to individuals in primary care, home, or telephone as necessary	Lay counsellors with at least secondary school education	(1) HAP and EUC; (2) EUC alone
Integrated interventions								
Carlson et al., 2012; [40] Witte et al., 2011 [41]	Mongolia	Cluster RCT	Adult female sex workers participating in NGO services who reported unprotected sex and screened positive for harmful alcohol use	166 (74 contributed to analysis)	HIV-STI Risk Reduction (HIV-SRR), a relationship-oriented and social cognitive theory-informed intervention including activities specific to protecting oneself from violence, enhanced with motivational interviewing (MI)	HIV-SRR: 4 90-min weekly sessions; MI2 weekly 90-min sessions; control: 4 weekly sessions, delivered to groups of 6–8 women	Females fluent in Mongol	(1) HIV-SSR; (2) HIV-SSR + MI (3) Control (wellness promotion training)
Jewkes et al., 2014 [38]	South Africa	Pre-post, interrupted time series	Community sample of adult men and women living in informal settlements characterized by extreme poverty; over 70% reported moderate or severe depression at baseline	232 (110 men, 122 women)	Creating Futures: a livelihood intervention aimed at strengthening use of resources in individuals’ environments, and *Stepping Stones*: a participatory learning intervention focused on preventing HIV transmission and fostering more equitable gender norms and inter-partner communication.	21 3-h sessions delivered 2x per week for 12 weeks to single sex groups of 20 individuals	Individuals of similar age to participants who completed secondary school	No control condition
Wechsberg et al., 2013 [42]	South Africa	RCT	Community sample of women living in disadvantaged areas who report using at least two drugs (one of which could be alcohol) once a week over the past 3-months	720 (688 contributed to analysis)	Women’s Health CoOp: an adapted women focused, empowerment-based HIV prevention intervention that also focuses on substance use and violence through use of role-playing, skill rehearsal, developing a risk-reduction plan, and provision of referrals. HIV counseling and testing (HCT).	2 1-h sessions delivered to groups of 4–6 women	Peer female educators	(1) WCH + HCT; (2) Nutrition + HCT; (3) HCT alone

#### Participants

Five studies (71%) included IPV survivors (three with female survivors [40–44], two mixed gender [39, 45, 46], one study included both IPV perpetrators (male) and IPV survivors (female) [38], and one study included IPV perpetrators (male) [47, 48]. Sample sizes ranged from 49 to 688, with the majority of studies (*n* = 5, 71%) including 200 or more participants. Two studies [38, 40, 41] had effective sample sizes of less than 100 (i.e., the number of IPV survivors or perpetrators within the broader study sample was < 100).

Two of the seven studies [40, 41, 43, 44] specifically focused on female sex workers, and two studies were conducted with disadvantaged communities, i.e. living in areas reserved for the use of “Black African” or “Coloured” persons systematically deprived under the Apartheid regime [42] and informal settlements [38] in South Africa. Two studies [45–48] were conducted in Indian primary care, and one study [39] included earthquake survivors in China.

#### Screening

Participants in four studies (57%) were screened into the study based on their scoring above a cut-off on a self-reported measure for harmful or hazardous alcohol use [40, 41, 43, 44, 47, 48] or moderately severe to severe depression [45]. One study screened in on the basis of meeting criteria for PTSD, depression, or both, using a structured psychiatric diagnostic interview [39]. One study applied a brief eligibility screening questionnaire to assess drug use eligibility criteria confirmed with biological testing [42]. One study did not screen for mental health problems, but found that 72% of the women and 75% of the men had moderate or severe depression symptoms [38].

#### IPV outcome measurement

All but one study (*n* = 6, 86%) assessed physical IPV [38, 40–48] and two studies each assessed sexual IPV [38, 40, 41] and psychological IPV or controlling behaviors [38, 45, 46]. One study reported all types of IPV combined using one measure [39]. None of the studies specifically noted that IPV was a primary outcome, and one study [38] did not distinguish primary vs secondary outcomes. Relatively few studies (*n* = 3, 43%) [39–42] used standardized measures such as the (Revised) Conflict Tactics Scale [49] or WHO Violence Against Women measures [50]. Rather, studies commonly used single-item survey questions (e.g., “In the past 3 months, have you slapped, hit, kicked, punched your wife/partner or done something else that did or could have hurt her physically?”) [47]. Almost all studies (*n* = 6, 86%) assessed change in IPV using a dichotomous score, commonly reported for the last three months, and most studies (*n* = 5, 71%) assessed IPV at 6 months and 12 months after intervention.

#### Mental health outcome measurement

Five studies (71%) [38, 40–44, 47, 48] focused on alcohol or drug use. All of these studies used the Alcohol Use Disorders Identification Test (AUDIT) to assess alcohol use, except Wechsberg and colleagues [42] who screened for alcohol use with a brief questionnaire and confirmed other drug use through biological testing on urine samples. Three studies (43%) focused on depression, using self-report questionnaires (two studies) [38, 45, 46] or a structured psychiatric diagnostic interview (one study) [39] The study that used the structured interview [39] also assessed PTSD using a psychiatric diagnostic interview.

#### Risk of bias

Overall, studies showed acceptable levels of risk of bias (Table 2). The most common issue (*n* = 5, 71%) concerned the lack of blinding of participants and personnel, which is challenging in mental health intervention studies. In addition, blinding of outcome assessment was not performed in three trials and it was unclear whether this was done in one study (total *n* = 4, 57%). Selective reporting did not appear to be a significant concern in any of the included studies: authors summarized results based on all included outcomes.

**Table 2:** Risk of bias of included studies

	Random sequence generation	Allocation concealment	Blinding participants/ personnel	Blinding outcome assessment	Incomplete outcome data	Selective reporting
Carlson 2012; [40] Witte 2011 [41]	Low	Low	High	Low	High	Low
Jewkes 2014 [38]	High	High	High	High	Low	Low
Jiang 2014 [39]	Low	Unclear	High	High	High	Low
L’Engle 2014 [43]; Parcesepe 2016 [44]	Low	Low	High	Unclear	Low	Low
Nadkarni 2017a,b [47, 48]	Low	Low	Low	Low	Low	Low
Patel 2017 [45]; Weobong 2017 [46]	Low	Low	Low	Low	Low	Low
Wechsberg 2013 [42]	Low	Unclear	High	High	Low	Low

### Characteristics of studied interventions

Interventions are summarized in Table 1, and study findings are summarized in Table 3.

**Table 3:** Narrative synthesis of study findings

Authors, year	Intervention category	Measure used	Outcome assessed	Effect
Carlson et al., 2012 [40]; Witte et al., 2011 [41]	Integrated intervention	Revised Conflict Tactics Scale (CTS2).	Victimization • Physical IPV (past 90 days) • Sexual IPV (past 90 days)	• 3 months post intervention: NS • 6 months post intervention: NS • 3 months post intervention: NS • 6 months post intervention: NS (*p*-values not reported)
Jewkes et al., 2014 [38]	Integrated intervention	WHO violence against women scale for all except sexual violence perpetration (asked did you ever force a girlfriend or wife into having sex with you). Dichotomized into any/none	Victimization (women) • Physical IPV (past 3 months) • Sexual IPV (past 3 months) Perpetration (men) • Physical IPV (past 3 months) • Sexual IPV (past 3 months)	• *p* = .12 • *p* = .03 – prevalence reduced from 30.3 to 18.9% • *p* = .49 • p = .69
Jiang et al., 2014 [39]	Dedicated mental health intervention (IPT)	Conflict Tactics Scale	Victimization (it appears for both men and women) • Combined psychological and physical (time frame not specified)	• 3 months post intervention: p-value not reported, Cohen’s *d* = −.38
L’Engle et al. 2014 [43] and Parcesepe et al., 2016 [44]	Dedicated mental health intervention (WHO brief alcohol reduction)	Asked “How many times in the last 30 days have you been beaten or physically abused?” dichotomized to any/none and report of having been forced to have sex against her desire yes/no	Victimization • Physical non-paying partner violence (past 30 days) • Sexual non-paying partner violence (past 30 days)	• 6 months post intervention: *p* = .09 • 12 months post intervention: *p* = .06 • 6 months post intervention: *p* = .69 • 12 months post intervention: p = .19
Nadkarni et al. 2017a [47] and Nadkarni et al. 2017b [48]	Dedicated mental health intervention (CAP- brief alcohol reduction)	Asked if the person had “slapped, hit, kicked, punched your wife/partner or done something else that did or could have hurt her physically.”	Perpetration (men) • Physical IPV (past 3 months)	• 3 months post intervention: *p* = .57 • 12 months post intervention: *p* = .28
Patel et al., 2017 [45] and Weobong et al. 2017 [46]	Dedicated mental health intervention (HAP)	2-items Physical: Has your husband/partner ever hit you?; Psychological: Has your husband/partner ever spoken to you using language which is threatening (for example that he is going to hit you) or abusive (called you names, accused you of having relations with other males, etc.)?	Victimization • Physical IPV • Psychological IPV	• 3 months post intervention physical (women): *p* = .04, aPR 0.53 (95% CI 0.29–0.96) • 12 months post intervention physical (women): *p* = .16 • 3 months post intervention physical (men): *p* = .24 • 3 months post intervention psychological (women): *p* = .15 • 12 months post intervention psychological (women): *p* = .17 • 3 months post intervention psychological (men): *p* = .41 • 12 months post intervention psychological (men): *p* = .62
Wechsberg et al., 2013 [42]	Integrated intervention	Asked about being slapped, pushed, shoved, kicked, hit, dragged, beaten, choked, or burned	• Physical IPV (past 6 months)	• 6 months post intervention: NS • 12 months post intervention: NS (*p*-values not reported)

#### Dedicated mental health treatments

Four of the seven studies (57%) [39, 43, 44, 47, 48] were dedicated mental health treatments, that is, they specifically targeted mental health through psychological interventions, with other outcomes considered secondary.

Two studies focused on common mental disorders. First, Jiang and colleagues [39] conducted a small pilot randomized controlled trial with adults (*n* = 41) meeting criteria for PTSD and/or depression two years following the 2008 Sichuan earthquake in China. Interpersonal psychotherapy (IPT) was selected based on qualitative data indicating that trauma-related symptoms were often linked to interpersonal difficulties, including spousal conflicts after children’s deaths. IPT was delivered by trained local personnel to individuals over 12 sessions.

Second, Patel [45], Weobong [46] and coworkers evaluated a brief psychological treatment (behavioral activation) with men and women (*n* = 495) screened for moderate to severe depression in primary care settings in Goa, India. The intervention was delivered by lay counselors over six to eight sessions and consisted of psychoeducation; behavioral assessment; activity monitoring, structuring, and scheduling; activation of social networks; and problem solving.

Two studies focused on substance abuse. First, L’Engle [43], Parcesepe [44] and coworkers implemented a randomized controlled trial comparing a brief alcohol-focused intervention with an equal-attention nutrition intervention control group. Participants were female adult sex workers (*n* = 818) with hazardous or harmful drinking patterns, recruited from drop-in centers in Mombasa, Kenya, of whom 565 had a non-paying partner. The intervention was based on the WHO Brief Intervention for Alcohol Use and consisted of six 20-min sessions of individual counseling, delivered approximately monthly by nurse counselors trained in motivational interviewing.

Second, Nadkarni and colleagues [47, 48] assessed impacts of a brief psychological treatment in a randomized controlled trial with male harmful drinkers (*n* = 377) in primary health care settings in Goa, India. Treatment was delivered individually by lay counsellors and included motivational interviewing, problem-solving, and general counselling strategies (e.g., open-ended questioning, showing empathy) combined with enhanced usual care. For participants who had a planned discharge in the treatment condition (70%), the number of sessions averaged 2.8 lasting 42 min.

#### Integrated interventions

Three of the seven studies (43%) [38, 40–42] tested integrated interventions in which a focus on mental health was combined with other intervention targets. Intervention aims included reducing risk for HIV/AIDS, reducing violence against particularly vulnerable women, and reducing substance abuse.

Witte [41], Carlson [40] and colleagues implemented a 3-armed randomized controlled trial to assess the impacts of an HIV/STI risk reduction intervention with adult female sex workers (*n* = 166) screened for harmful alcohol use in Mongolia. The sexual risk reduction intervention was based on social cognitive and ecological theory [51, 52] and consisted of four sessions with a relationship focus (the relationship with the paying sexual partner). It included information on how to protect oneself from violence (not IPV-specific). This intervention was tested with and without two wrap-around sessions of motivational interviewing aimed at reducing harmful alcohol use.

Jewkes and coworkers [38] conducted a shortened interrupted time series to pilot an HIV-and violence prevention intervention (Stepping Stones, 10 group sessions) followed by a livelihoods-focused intervention (Creating Futures, 11 group sessions) implemented in 3-h bi-weekly sessions over 12 weeks. Participants were young men and women (*n* = 232, mostly aged between 18 and 30) living in informal settlements in Durban, South Africa, over 70% of whom reported moderate or severe depression at baseline. Stepping Stones is based on participatory learning approaches, including critical reflection, role play, and drama. It consists of 10 3-h sessions with single-sex groups, who come together to discuss learning on more gender equitable relationships and improved communication. Participants also discuss motivations for behavior, including influences of alcohol and poverty.

Wechsberg and colleagues [42] conducted a 3-armed randomized controlled trial, comparing impacts of (1) the Women’s Health CoOp adapted for use in Pretoria, South Africa, together with HIV testing and counseling; (2) a nutrition intervention with HIV testing and counseling; and (3) HIV testing and counseling alone. The Women’s Health CoOp is a two-session intervention delivered by peer educators who provide information about drug use and sex-risk behavior, and practice skills with groups of 4–6 women. Of the four 1-h modules, one is focused on information about drug use and risks. Sessions also focus on skills to negotiate condom use and avoiding potentially violent situations. Participants were women of child-bearing age (*n* = 720) living in disadvantaged Cape Town communities who reported at least weekly use of two types of drugs (one could be alcohol) in the last three months.

### Impacts of interventions

#### Dedicated mental health treatments

Mental health benefits and reduction in alcohol misuse were consistently found across the dedicated mental health treatments, but reductions in IPV were inconsistently identified. Two of the dedicated mental health treatments focused on depression. Despite the small sample, Jiang and colleagues [39] identified reductions in PTSD and depression diagnoses three months after interpersonal therapy with earthquake-affected adults in China (generalized equation estimates B = 2.37, *p* = 0.018 PTSD and; B = 1.91, *p* = 0.56 for depression). They also identified reductions on a combined IPV measure in both men and women, reflecting both perpetration and victimization (Cohen’s d = −.38). Second, Patel, [45] Weobong [46] and coworkers found that behavioral activation was associated with reductions on depression symptom severity and remission at both 3- and 12-month assessments in primary care centers in India. Although physical IPV victimization in women was reduced in the treatment arm at three months (adjusted mean difference [aMD] 0.53, *p* = 0.04), this difference was not maintained at 12 months. No differences were found for women’s psychological IPV victimization, or men’s physical and psychological IPV victimization.

The other two dedicated mental health treatments focused on alcohol misuse. L’Engle [43], Parcesepe [44] and colleagues identified consistent benefits for a 6-session motivational interviewing with female sex workers in Kenya across the alcohol-related outcomes at both 6- and 12-month assessments (frequency of drinking 12-month adjusted odds ratio [aOR] = .25, *p* < .0001; binge drinking aOR = .18 p < .0001, binge drinking before sex with non-paying partner aOR = .26, *p* = .0002). However, no differences between study arms were identified for sexual IPV victimization (12-month aOR = .76, *p* = .19). Nadkarni et al. [47, 48] studied the benefits of a motivational interviewing-based intervention with male problem drinkers in primary care in Goa, India, and found benefits at 12 months for remission (adjusted prevalence ratio 1.71, *p* < .001) and abstinence (aOR = 1.92, *p* = .008), but no impacts on IPV perpetration were identified.

#### Integrated interventions

The three integrated interventions showed mixed results for mental health and IPV outcomes (Table 3). A study with female sex workers in Mongolia [41] found no difference in alcohol use reduction across the three study arms (i.e., HIV/STI risk reduction intervention, the HIV/STI risk reduction intervention enhanced with two wraparound sessions of motivational interviewing, and control arm). There was also no difference in the incidence of physical IPV and sexual IPV victimization [40]. Because of low shares of participants reporting an intimate partner (39 to 51%), between-group statistical testing was not conducted separately for intimate partner and paying partner-perpetrated violence. The Women’s Health CoOp intervention with vulnerable drug-using women in South Africa [42] did result in significantly greater improvements in drug abstinence in the intervention than the combined control arms (Odds Ratio [OR] = 1.54, Confidence Interval [IC] = 1.07–2.22, Cohen’s d effect size = 0.238). However, there were no statistically significant differences in physical IPV victimization, which declined in all three study arms.

Jewkes and colleagues’ [38] pilot of a combined HIV/violence prevention and livelihoods-focused intervention identified gendered mental health and IPV benefits. Interrupted time series analyses identified reductions in moderate to severe depression and suicidal thoughts for men (75 to 53% and 26 to 10%, respectively), but not women. In addition, women’s problematic alcohol use increased (27 to 36%). No changes were identified for men’s alcohol or other drug use, nor women on other drug use. Regarding IPV perpetration by men, small but significant reductions in controlling behaviors (19 to 22%) were found, but no statistically significant reductions in physical, sexual, or combined physical/sexual IPV perpetration were identified. Statistically significant reductions were observed in women’s experience of sexual IPV and combined sexual/ physical IPV (10 to 4% and 30 to 9%, respectively), but this was not the case for experience of controlling behaviors or physical IPV.

## Discussion

This systematic review aimed to synthesize findings from controlled studies in LMIC of the impact of mental health treatments on the prevention and reduction of IPV. Despite our search across a wide range of databases and screening more than 1000 titles and abstracts, we found only seven studies that have evaluated the benefits of mental health treatments with regard to IPV in LMIC. These studies were conducted in five middle-income countries. Six were RCTs and one had an interrupted time series design. The research topic addressed here does seem to be an area of increasing interest: as part of our searches we identified 10 published protocols for planned or ongoing trials that likely meet inclusion criteria once completed (available upon request).

The main observation from this review is that the current literature is limited in scope, resulting in critical gaps in our knowledge. In our view, the main overall gaps concern: (1) no studies have replicated evaluations of similar treatments; (2) no studies were conducted in low-income countries; (3) there was a lack of diverse samples (e.g., no studies in humanitarian settings or with refugee populations, where rates of IPV are particularly high [53]; no studies with older adolescents; no studies with sexual minorities); (4) limited geographical coverage (e.g., no studies from Latin America and the Caribbean, Middle East and North Africa, West Africa); and (5) a limited range of mental health concerns were targeted (e.g., no studies focused on anger dysregulation or medically unexplained complaints).

### Benefits of mental health interventions for IPV outcomes

Though limited in number, studies evaluated both integrated interventions (*n* = 3) and dedicated mental health treatments (*n* = 4). Integrated interventions were aimed at concurrently reducing HIV/STI risk, violence against women, and alcohol or other substance misuse treatment, whereas dedicated mental health treatments targeted common mental disorders (depression, PTSD) or alcohol misuse. As the driving hypothesis for assessing the impact of these interventions on IPV is that mental health concerns are a possible pathway through which IPV perpetration or victimization can be stopped, reduced, or prevented, it should be noted that the studies included in our review produced mixed findings in relation to mental health outcomes. In evaluations of three integrated interventions, one [40, 41] produced null findings for harmful alcohol use. In the second, improvements were found for men’s depression and suicidal thoughts, but alcohol use increased in women [38]. In the third [42] reductions in drug use were identified.

Somewhat more promising findings were identified for the four dedicated mental health treatments: all four showed impacts on mental health outcomes. Of the dedicated mental health treatments, the two studies that focused on depression also identified reductions in IPV: one found reductions for a combined perpetration/ victimization measure in men and women, and one found reduced IPV victimization in women [39, 45] These benefits were not maintained at 12 months in one of the studies [46]. The two studies that focused on alcohol misuse did not identify benefits with regard to IPV victimization or perpetration [43, 44, 47, 48].

### Recommendations

It is challenging to draw firm conclusions from this limited pool of studies. In this regard, our review echoes findings from previous broader reviews of interventions to prevent or reduce violence against women and girls, which note the lack of literature particularly from LMIC [54, 55]. The ‘What Works to Prevent Violence’ program’s synthesis of existing knowledge [55] specifically highlights a knowledge gap with regard to the impact of mental health on perpetration and experience of violence. Further research is urgently needed. Based on the existing studies, we believe several research strategies would improve research on this topic as it develops.

#### Strengthen theoretical underpinnings

Critically, research on this topic would benefit from stronger theoretical development. This is because existing studies commonly did not detail the specific pathways through which improvements of mental health conditions were hypothesized to impact IPV perpetration or victimization. In the case of dedicated mental health interventions, IPV was included as a secondary outcome. More explicit thought regarding intervention adaptation and study design for the specific question of whether mental health interventions can address IPV is an important step to developing evidence better geared for answering this question. Several ongoing studies have begun to do this [56, 57]. For example, one study with IPV survivors is examining whether improved social support, coping, and support seeking as a result from a group psychological intervention is associated with subsequent reductions in IPV victimization [57]. Similarly, as noted in the introduction, evaluations of mental health interventions may assess whether changes in self-esteem, self-blame, or emotional numbing lead to reductions in IPV victimization, or whether improvements in managing strong emotions are associated with reductions in IPV perpetration.

The few benefits identified in the current literature suggest treatment strategies and/or pathways that could be targeted for more explicit study and replication. E.g., Patel, Weobong and colleagues identified reductions in physical IPV victimization amongst women after a behavioral intervention at immediate post-treatment follow-up, but not at the 12-month follow-up. Similarly, Jiang and coworkers’ small pilot trial (2014) found impacts on depression and PTSD and reduced interpersonal therapy on a combined measure of IPV perpetration and victimization in male and female earthquake survivors. They hypothesized a mental health and IPV connection based on formative qualitative research that indicated the disaster had heightened interpersonal conflicts among affected couples. This study points to how mixed-methods research may strengthen conceptual models for future testing of complex interventions [58].

Building on improvements in conceptualizations of pathways, research on this topic would benefit from directly testing hypothesized relationships between mental health and IPV perpetration or victimization. This can, for example, be achieved through by measuring treatment effects on potential mediating variables, where reductions on mental health variables can be statistically assessed for correlation with subsequent reductions in IPV. This will be particularly important for complex interventions, where multiple components may explain prevention or reduction of IPV. In addition, theoretical models may be tested through more complex trial designs, e.g. trials with arms including combined mental health and violence protection activities, mental health and violence protection alone, against a control condition.

#### Improve IPV measurement

A major limitation of the current body of studies is that they may have been underpowered to detect changes in IPV. This may partly be due to the common use of dichotomous rather than continuous outcome measures for IPV. In addition, power in the existing studies was likely reduced because studies were not specifically geared to addressing changes on IPV, using samples in which IPV was present only in subsamples. IPV was commonly included as a secondary outcome measure, with studies only specifically powered to detect changes on primary outcomes. In addition, a few studies used non-standardized measures for IPV with unknown psychometric properties, including sensitivity to change.

#### Strengthen mental health benefits in integrated interventions

The limited mental health impacts of the integrated interventions raise important questions for future studies. The lack of clear mental health benefits from the integrated interventions (one out of three studies), may be an issue of content or dose (e.g., number of hours) of specific mental health-focused content. In integrated interventions, this dose may be smaller given that other content within the integrated intervention may target social determinants of poor mental health (i.e., IPV, social isolation, and poverty). At the same time, increasing the amount of mental health related content needs to be weighed against feasibility concerns in low-resource settings. To ensure feasibility, ongoing efforts are required to improve the scalability of existing evidence based mental health interventions in LMIC. For example, transdiagnostic interventions, by combining treatment techniques for commonly comorbid mental health concerns, reduce the number of protocols for training health workers, making it more attractive for agencies not specialized in mental health to introduce mental health interventions as part of their work [59, 60]. Similarly, alternative intervention formats (e.g. electronic delivery or self-help formats) may increase feasibility and thereby uptake by non-specialized agencies [61].

#### Consider promising strategies from HIC

Several promising strategies from HIC are also worth exploring in LMIC. For example, a more narrow systematic review of cognitive behavioral and advocacy interventions (which commonly have psychosocial support elements) with IPV survivors in high-income settings identified 12 randomized controlled trials, and found that both showed impacts on physical and psychological but not sexual and combined IPV victimization [62]. With regard to IPV perpetrators, there is limited evidence from HIC on cognitive behavioral interventions for male perpetrators of physical IPV [18]. In addition, an emerging literature in high-income settings has found promising results for innovative approaches, e.g. for couples in which male perpetrators receive treatment for anger dysregulation, alcohol misuse, and/ or common mental disorders [19, 20]. IPV perpetration interventions may specifically target people at higher risk for perpetration, e.g. men with histories of childhood trauma and current anger dysregulation concerns, including harsh parenting [63].

### Limitations of systematic review

Our a priori aim was to include studies focused on reducing (symptoms of) mental disorders (i.e., studies focused on treatments evaluated with populations screened to have higher levels of symptoms or meeting criteria for disorders). Mental health is more than the absence of symptoms, and positive aspects of mental health may be targeted as protective factors against IPV. For example, positive parenting practices during childhood may be associated with reduced IPV perpetration in adulthood [64]. Inclusion of studies focused on promoting positive aspects of mental health may have resulted in the identification of studies dedicated to the primary prevention of IPV. In addition, at the title and abstract screening phase we only included studies with an abstract in English.

## Conclusions

We identified a limited number of studies evaluating the impact of mental health treatments on the prevention or reduction of IPV in LMIC, despite epidemiological research suggesting that this would be a potentially beneficial direction for research and intervention. Existing studies are of reasonable quality, but more studies are required in diverse settings and with diverse populations that explicitly are designed to address this research question. Specifically, future research on this topic would benefit from stronger theoretical development and designs aimed at disentangling the benefits of targeting mental health-related risk and protective factors within the wider constellation of IPV determinants across the socio-ecological system. Studies in LMIC may benefit from promising emerging findings in HIC, and integrated interventions may build on innovative efforts to improve the scalability of evidence-based mental health interventions in LMIC.

## Additional file

Additional file 1: MH-IPV Search Strategy for PubMed. (DOCX 18 kb)

## Abbreviations

aMD: Adjusted mean difference
aOR: Adjusted odds ratio
HIC: High-income countries
HIV/AIDS: Human Immunodeficiency Virus/Acquired Immune Deficiency Syndrome
IPT: Interpersonal psychotherapy
IPV: Intimate partner violence
LMIC: Low- and middle-income countries
MeSH: Medical subject headings
PTSD: Posttraumatic Stress Disorder
RCT: Randomized controlled trial
STI: Sexually transmitted infections
WHO: World Health Organization
