# Supplementary material for: Can mental health treatments help prevent or reduce intimate partner violence in low- and middle-income countries? A systematic review
Source: BMC Womens Health. 2019 Feb 14;19:34. doi: 10.1186/s12905-019-0728-z (PMC6376658; doi:10.1186/s12905-019-0728-z)
Supplement: Supplementary file 1 — MH-IPV Search Strategy for PubMed. (DOCX 18 kb) [file 12905_2019_728_MOESM1_ESM.docx]

**MH-IPV Search Strategy for PubMed**

Searched: June 26^th^, 2017-06-26

Number of Articles: 335

**Outcome**

(“intimate partner violence”[mesh] OR domestic violence[mesh:noexp]

OR “partner abuse”[tw] OR “relationship aggression”[tw] OR “domestic abuse”[tw] OR “marital abuse”[tw] OR “spousal abuse”[tw] OR "spouse abuse"[tw] OR “wife beating”[tw] OR “intimate terrorism”[tw] OR “marital rape”[tw] OR “battered women”[tw] OR “abused women”[tw] OR “rape”[tw] OR “psychological abuse”[tw] OR “reproductive coercion”[tw] OR "violence"[tw])

**Intervention Type**

AND ("Affective Symptoms"[Mesh] OR "Aggression"[Majr:noexp] OR "Alcohol Drinking"[Mesh] OR "Anxiety"[Mesh] OR "Depression"[Mesh] OR "Diagnostic and Statistical Manual of Mental Disorders"[Mesh] OR "Epilepsy"[Mesh] OR "Impulsive Behavior"[Mesh] OR "Irritable Mood"[Mesh] OR "Mental Disorders"[Mesh] OR "Mental Fatigue"[Mesh] OR "Mentally Ill Persons"[Mesh] OR "Paranoid Behavior"[Mesh] OR "Problem Behavior"[Mesh] OR "Psychophysiologic Disorders"[Mesh] OR "Self-Injurious Behavior"[Mesh] OR "Stress, Psychological"[Mesh] OR "Mental Health"[Mesh] OR Addiction[Text Word] OR Alcohol abuse[Text Word] OR Alcohol use Disorder[Text Word] OR Anxiety[Text Word] OR Attention Deficit disorder[Text Word] OR Bipolar[Text Word] OR Child Behaviour Disorder[Text Word] OR Child Development Disorder[Text Word] OR Conduct Disorder[Text Word] OR Depression[Text Word] OR Depressive Disorder[Text Word] OR Developmental Disability[Text Word] OR Emotional stress[Text Word] OR Epilepsies[Text Word] OR Epilepsy[Text Word] OR Mania[Text Word] OR Mental disorder[Text Word] OR Mental illness[Text Word] OR Mental stress[Text Word] OR Mentally ill[Text Word] OR Mood Disorder[Text Word] OR Neurodevelopmental Disorder[Text Word] OR Personality Disorder[Text Word] OR Posttraumatic stress disorder[Text Word] OR Post-traumatic stress disorder[Text Word] OR Psychiatric[Text Word] OR Psychological distress[Text Word] OR Psychological stress[Text Word] OR Psychological trauma[Text Word] OR Psychosis[Text Word] OR Psychoses[Text Word] OR Psychotic[Text Word] OR psychosomatic[Text Word] OR Schizophrenia[Text Word] OR Schizophrenic[Text Word] OR Somatisation[Text Word] OR Somatoform[Text Word] OR Stress Disorder[Text Word] OR Substance abuse[Text Word] OR Substance Disorder[Text Word] OR Substance Withdrawal Syndrome[Text Word] OR Suicidal[Text Word] OR Traumatic stress disorder[Text Word] OR mental health problems[Text Word] OR common mental disorders[Text Word] OR Mental disorders[Text Word] OR Mental Health[Text Word] OR cocaine[Text Word] OR heroin[Text Word])

**Intervention Strategy**

AND ("Caregivers"[Mesh] OR "Combined Modality Therapy"[Mesh] OR "Community Health Workers"[Mesh] OR "Comprehensive Health Care"[Mesh] OR "Health Education"[Majr:noexp] OR "Hospitals, Psychiatric"[Mesh] OR "Hospitals, University"[Mesh] OR "Interview, Psychological"[Mesh] OR "Mental Health Services"[Mesh] OR "Psychiatric Somatic Therapies"[Mesh] OR "Psychoanalytic Interpretation"[Mesh] OR "Psychological Techniques"[Mesh] OR "Psychopharmacology"[Mesh] OR "Psychotherapy"[Mesh] OR "Psychotropic Drugs"[Mesh] OR "Psychotropic Drugs"[Pharmacological Action] OR "Self-Help Groups"[Mesh] OR "Ambulatory Care Facilities"[Mesh] OR Therapy[Text Word] OR psychotherapy[Text Word] OR mental health care[Text Word] OR mental health services[Text Word] OR mental health service[Text Word] OR psychotherapeutic[Text Word] OR addiction recovery[Text Word] OR counselling[Text Word] OR counseling[Text Word] OR behavioral activation[Text Word] OR behavioural activation[Text Word] OR behavioural management[Text Word] OR behavioral management[Text Word] OR contingency management[Text Word] OR epilepsy surgery[Text Word] OR eye movement desensitization[Text Word] OR hypnosis[Text Word] OR prolonged exposure[Text Word] OR locus of control[Text Word] OR mindfulness[Text Word] OR motivational interview[Text Word] OR neurolinguistic programming[Text Word] OR pastoral care[Text Word] OR psychoanalytic[Text Word] OR psychodynamic[Text Word] OR psychoeducation[Text Word] OR psychological feedback[Text Word] OR psychological interview[Text Word] OR relaxation training[Text Word] OR self affirmation[Text Word] OR supported employment[Text Word] OR psychotropic drugs[Text Word] OR psychotropic drug[Text Word] OR psychopharmacology[Text Word] OR psychotropic[Text Word] OR antipsychotic[Text Word] OR antidepressant[Text Word] OR anxiolytic[Text Word] OR antianxiety[Text Word] OR mood stabilizer[Text Word] OR mood stabiliser[Text Word] OR treatment[Text Word] OR intervention[Text Word] OR CBT[Text Word] OR MBCT[Text Word] OR cognitive therapy[Text Word] OR interpersonal[Text Word] OR Rehabilitation[Text Word] OR psychiatric hospital[Text Word] OR drug treatment[Text Word] OR psychological treatment[Text Word] OR psychoeducational[Text Word] OR Family-based[Text Word] OR solution-focused[Text Word] OR brief[Title] OR carers[Text Word] OR caregivers[Text Word] OR community-based[Text Word] OR behaviour therapy[Text Word] OR behavior therapy[Text Word] OR encounter groups[Text Word] OR preventive health care[Text Word] OR preventive programs[Text Word] OR preventive programmes[Text Word] OR preventive health service[Text Word] OR mental hospital[Text Word] OR mental institution[Text Word] OR mental health education[Text Word] OR sensitivity training[Text Word] OR teaching hospital[Text Word])

**Population**

AND (Africa[Title/Abstract] OR Asia[Title/Abstract] OR Caribbean[Title/Abstract] OR West Indies[Title/Abstract] OR South America[Title/Abstract] OR Latin America[Title/Abstract] OR Central America[Title/Abstract] OR "Atlantic Islands"[Title/Abstract] OR "Commonwealth of Independent States"[Title/Abstract] OR "Pacific Islands"[Title/Abstract] OR "Indian Ocean Islands"[Title/Abstract] OR "Eastern Europe"[Title/Abstract] OR Afghanistan[Title/Abstract] OR Albania[Title/Abstract] OR Algeria[Title/Abstract] OR Angola[Title/Abstract] OR Antigua[Title/Abstract] OR Barbuda[Title/Abstract] OR Argentina[Title/Abstract] OR Armenia[Title/Abstract] OR Armenian[Title/Abstract] OR Aruba[Title/Abstract] OR Azerbaijan[Title/Abstract] OR Bahrain[Title/Abstract] OR Bangladesh[Title/Abstract] OR Barbados[Title/Abstract] OR Benin[Title/Abstract] OR Byelarus[Title/Abstract] OR Byelorussian[Title/Abstract] OR Belarus[Title/Abstract] OR Belorussian[Title/Abstract] OR Belorussia[Title/Abstract] OR Belize[Title/Abstract] OR Bhutan[Title/Abstract] OR Bolivia[Title/Abstract] OR Bosnia[Title/Abstract] OR Herzegovina[Title/Abstract] OR Hercegovina[Title/Abstract] OR Botswana[Title/Abstract] OR Brasil[Title/Abstract] OR Brazil[Title/Abstract] OR Bulgaria[Title/Abstract] OR Burkina Faso[Title/Abstract] OR Burkina Fasso[Title/Abstract] OR Upper Volta[Title/Abstract] OR Burundi[Title/Abstract] OR Urundi[Title/Abstract] OR Cambodia[Title/Abstract] OR Khmer Republic[Title/Abstract] OR Kampuchea[Title/Abstract] OR Cameroon[Title/Abstract] OR Cameroons[Title/Abstract] OR Cameron[Title/Abstract] OR Cameroun[Title/Abstract] OR Cape Verde[Title/Abstract] OR Central African Republic[Title/Abstract] OR Chad[Title/Abstract] OR Chile[Title/Abstract] OR China[Title/Abstract] OR Colombia[Title/Abstract] OR Comoros[Title/Abstract] OR Comoro Islands[Title/Abstract] OR Comores[Title/Abstract] OR Mayotte[Title/Abstract] OR Congo[Title/Abstract] OR Zaire[Title/Abstract] OR Costa Rica[Title/Abstract] OR Cote d'Ivoire[Title/Abstract] OR Ivory Coast[Title/Abstract] OR Croatia[Title/Abstract] OR Cuba[Title/Abstract] OR Cyprus[Title/Abstract] OR Czechoslovakia[Title/Abstract] OR Czech Republic[Title/Abstract] OR Slovakia[Title/Abstract] OR Slovak Republic[Title/Abstract] OR Djibouti[Title/Abstract] OR French Somaliland[Title/Abstract] OR Dominica[Title/Abstract] OR Dominican Republic[Title/Abstract] OR East Timor[Title/Abstract] OR East Timur[Title/Abstract] OR Timor Leste[Title/Abstract] OR Ecuador[Title/Abstract] OR Egypt[Title/Abstract] OR United Arab Republic[Title/Abstract] OR El Salvador[Title/Abstract] OR Eritrea[Title/Abstract] OR Estonia[Title/Abstract] OR Ethiopia[Title/Abstract] OR Fiji[Title/Abstract] OR Gabon[Title/Abstract] OR Gabonese Republic[Title/Abstract] OR Gambia[Title/Abstract] OR Gaza[Title/Abstract] OR Georgia Republic[Title/Abstract] OR Georgian Republic[Title/Abstract] OR Ghana[Title/Abstract] OR Gold Coast[Title/Abstract] OR Greece[Title/Abstract] OR Grenada[Title/Abstract] OR Guatemala[Title/Abstract] OR Guinea[Title/Abstract] OR Guam[Title/Abstract] OR Guiana[Title/Abstract] OR Guyana[Title/Abstract] OR Haiti[Title/Abstract] OR Honduras[Title/Abstract] OR Hungary[Title/Abstract] OR India[Title/Abstract] OR Maldives[Title/Abstract] OR Indonesia[Title/Abstract] OR Iran[Title/Abstract] OR Iraq[Title/Abstract] OR Isle of Man[Title/Abstract] OR Jamaica[Title/Abstract] OR Jordan[Title/Abstract] OR Kazakhstan[Title/Abstract] OR Kazakh[Title/Abstract] OR Kenya[Title/Abstract] OR Kiribati[Title/Abstract] OR Korea[Title/Abstract] OR Kosovo[Title/Abstract] OR Kyrgyzstan[Title/Abstract] OR Kirghizia[Title/Abstract] OR Kyrgyz Republic[Title/Abstract] OR Kirghiz[Title/Abstract] OR Kirgizstan[Title/Abstract] OR "Lao PDR"[Title/Abstract] OR Laos[Title/Abstract] OR Latvia[Title/Abstract] OR Lebanon[Title/Abstract] OR Lesotho[Title/Abstract] OR Basutoland[Title/Abstract] OR Liberia[Title/Abstract] OR Libya[Title/Abstract] OR Lithuania[Title/Abstract] OR Macedonia[Title/Abstract] OR Madagascar[Title/Abstract] OR Malagasy Republic[Title/Abstract] OR Malaysia[Title/Abstract] OR Malaya[Title/Abstract] OR Malay[Title/Abstract] OR Sabah[Title/Abstract] OR Sarawak[Title/Abstract] OR Malawi[Title/Abstract] OR Nyasaland[Title/Abstract] OR Mali[Title/Abstract] OR Malta[Title/Abstract] OR Marshall Islands[Title/Abstract] OR Mauritania[Title/Abstract] OR Mauritius[Title/Abstract] OR Agalega Islands[Title/Abstract] OR "Melanesia"[Title/Abstract] OR Mexico[Title/Abstract] OR Micronesia[Title/Abstract] OR Middle East[Title/Abstract] OR Moldova[Title/Abstract] OR Moldovia[Title/Abstract] OR Moldovian[Title/Abstract] OR Mongolia[Title/Abstract] OR Montenegro[Title/Abstract] OR Morocco[Title/Abstract] OR Ifni[Title/Abstract] OR Mozambique[Title/Abstract] OR Myanmar[Title/Abstract] OR Myanma[Title/Abstract] OR Burma[Title/Abstract] OR Namibia[Title/Abstract] OR Nepal[Title/Abstract] OR Netherlands Antilles[Title/Abstract] OR New Caledonia[Title/Abstract] OR Nicaragua[Title/Abstract] OR Niger[Title/Abstract] OR Nigeria[Title/Abstract] OR Northern Mariana Islands[Title/Abstract] OR Oman[Title/Abstract] OR Muscat[Title/Abstract] OR Pakistan[Title/Abstract] OR Palau[Title/Abstract] OR Palestine[Title/Abstract] OR Panama[Title/Abstract] OR Paraguay[Title/Abstract] OR Peru[Title/Abstract] OR Philippines[Title/Abstract] OR Philipines[Title/Abstract] OR Phillipines[Title/Abstract] OR Phillippines[Title/Abstract] OR Poland[Title/Abstract] OR Portugal[Title/Abstract] OR Puerto Rico[Title/Abstract] OR Romania[Title/Abstract] OR Rumania[Title/Abstract] OR Roumania[Title/Abstract] OR Russia[Title/Abstract] OR Russian[Title/Abstract] OR Rwanda[Title/Abstract] OR Ruanda[Title/Abstract] OR Saint Kitts[Title/Abstract] OR St Kitts[Title/Abstract] OR Nevis[Title/Abstract] OR Saint Lucia[Title/Abstract] OR St Lucia[Title/Abstract] OR Saint Vincent[Title/Abstract] OR St Vincent[Title/Abstract] OR Grenadines[Title/Abstract] OR Samoa[Title/Abstract] OR Samoan Islands[Title/Abstract] OR Navigator Island[Title/Abstract] OR Navigator Islands[Title/Abstract] OR Sao Tome[Title/Abstract] OR Saudi Arabia[Title/Abstract] OR Senegal[Title/Abstract] OR Serbia[Title/Abstract] OR Montenegro[Title/Abstract] OR Seychelles[Title/Abstract] OR Sierra Leone[Title/Abstract] OR Slovenia[Title/Abstract] OR Sri Lanka[Title/Abstract] OR Ceylon[Title/Abstract] OR Solomon Islands[Title/Abstract] OR Somalia[Title/Abstract] OR Sudan[Title/Abstract] OR South Sudan[Title/Abstract] OR South Africa[Title/Abstract] OR Suriname[Title/Abstract] OR Surinam[Title/Abstract] OR Swaziland[Title/Abstract] OR Syria[Title/Abstract] OR Syrian[Title/Abstract] OR Tajikistan[Title/Abstract] OR Tadzhikistan[Title/Abstract] OR Tadjikistan[Title/Abstract] OR Tadzhik[Title/Abstract] OR Tanzania[Title/Abstract] OR Thailand[Title/Abstract] OR Togo[Title/Abstract] OR Togolese Republic[Title/Abstract] OR Tonga[Title/Abstract] OR Trinidad[Title/Abstract] OR Tobago[Title/Abstract] OR Tunisia[Title/Abstract] OR Turkey[Title/Abstract] OR Turkmenistan[Title/Abstract] OR Turkmen[Title/Abstract] OR Tuvalu[Title/Abstract] OR Uganda[Title/Abstract] OR Ukraine[Title/Abstract] OR Uruguay[Title/Abstract] OR USSR[Title/Abstract] OR Soviet Union[Title/Abstract] OR Union of Soviet Socialist Republics[Title/Abstract] OR Uzbekistan[Title/Abstract] OR Uzbek OR Vanuatu[Title/Abstract] OR New Hebrides[Title/Abstract] OR Venezuela[Title/Abstract] OR Vietnam[Title/Abstract] OR Viet Nam[Title/Abstract] OR West Bank[Title/Abstract] OR Yemen[Title/Abstract] OR Yugoslavia[Title/Abstract] OR Zambia[Title/Abstract] OR Zimbabwe[Title/Abstract] OR Rhodesia[Title/Abstract] OR "developing country"[Title/Abstract] OR "developing countries"[Title/Abstract] OR "developing nation"[Title/Abstract] OR "developing nations"[Title/Abstract] OR "developing population"[Title/Abstract] OR "developing populations"[Title/Abstract] OR "developing world"[Title/Abstract] OR "less developed country"[Title/Abstract] OR "less developed countries"[Title/Abstract] OR "less developed nation"[Title/Abstract] OR "less developed nations"[Title/Abstract] OR "less developed population"[Title/Abstract] OR "less developed populations"[Title/Abstract] OR "less developed world"[Title/Abstract] OR "lesser developed country"[Title/Abstract] OR "lesser developed countries"[Title/Abstract] OR "lesser developed nation"[Title/Abstract] OR "lesser developed nations"[Title/Abstract] OR "lesser developed population"[Title/Abstract] OR "lesser developed populations"[Title/Abstract] OR "lesser developed world"[Title/Abstract] OR "under developed country"[Title/Abstract] OR "under developed countries"[Title/Abstract] OR "under developed nation"[Title/Abstract] OR "under developed nations"[Title/Abstract] OR "under developed population"[Title/Abstract] OR "under developed populations"[Title/Abstract] OR "under developed world"[Title/Abstract] OR "underdeveloped country"[Title/Abstract] OR "underdeveloped countries"[Title/Abstract] OR "underdeveloped nation"[Title/Abstract] OR "underdeveloped nations"[Title/Abstract] OR "underdeveloped population"[Title/Abstract] OR "underdeveloped populations"[Title/Abstract] OR "underdeveloped world"[Title/Abstract] OR "middle income country"[Title/Abstract] OR "middle income countries"[Title/Abstract] OR "middle income nation"[Title/Abstract] OR "middle income nations"[Title/Abstract] OR "middle income population"[Title/Abstract] OR "middle income populations"[Title/Abstract] OR "low income country"[Title/Abstract] OR "low income countries"[Title/Abstract] OR "low income nation"[Title/Abstract] OR "low income nations"[Title/Abstract] OR "low income population"[Title/Abstract] OR "low income populations"[Title/Abstract] OR "lower income country"[Title/Abstract] OR "lower income countries"[Title/Abstract] OR "lower income nation"[Title/Abstract] OR "lower income nations"[Title/Abstract] OR "lower income population"[Title/Abstract] OR "lower income populations"[Title/Abstract] OR "underserved country"[Title/Abstract] OR "underserved countries"[Title/Abstract] OR "underserved nation"[Title/Abstract] OR "underserved nations"[Title/Abstract] OR "underserved population"[Title/Abstract] OR "underserved populations"[Title/Abstract] OR "underserved world"[Title/Abstract] OR "under served country"[Title/Abstract] OR "under served countries"[Title/Abstract] OR "under served nation"[Title/Abstract] OR "under served nations"[Title/Abstract] OR "under served population"[Title/Abstract] OR "under served populations"[Title/Abstract] OR "under served world"[Title/Abstract] OR "deprived country"[Title/Abstract] OR "deprived countries"[Title/Abstract] OR "deprived nation"[Title/Abstract] OR "deprived nations"[Title/Abstract] OR "deprived population"[Title/Abstract] OR "deprived populations"[Title/Abstract] OR "deprived world"[Title/Abstract] OR "poor country"[Title/Abstract] OR "poor countries"[Title/Abstract] OR "poor nation"[Title/Abstract] OR "poor nations"[Title/Abstract] OR "poor population"[Title/Abstract] OR "poor populations"[Title/Abstract] OR "poor world"[Title/Abstract] OR "poorer country"[Title/Abstract] OR "poorer countries"[Title/Abstract] OR "poorer nation"[Title/Abstract] OR "poorer nations"[Title/Abstract] OR "poorer population"[Title/Abstract] OR "poorer populations"[Title/Abstract] OR "poorer world"[Title/Abstract] OR "developing economy"[Title/Abstract] OR "developing economies"[Title/Abstract] OR "less developed economy"[Title/Abstract] OR "less developed economies"[Title/Abstract] OR "lesser developed economy"[Title/Abstract] OR "lesser developed economies"[Title/Abstract] OR "under developed economy"[Title/Abstract] OR "under developed economies"[Title/Abstract] OR "underdeveloped economy"[Title/Abstract] OR "underdeveloped economies"[Title/Abstract] OR "middle income economy"[Title/Abstract] OR "middle income economies"[Title/Abstract] OR "low income economy"[Title/Abstract] OR "low income economies"[Title/Abstract] OR "lower income economy"[Title/Abstract] OR "lower income economies"[Title/Abstract] OR "low gdp"[Title/Abstract] OR "low gnp"[Title/Abstract] OR "low gross domestic"[Title/Abstract] OR "low gross national"[Title/Abstract] OR "lower gdp"[Title/Abstract] OR "lower gnp"[Title/Abstract] OR "lower gross domestic"[Title/Abstract] OR "lower gross national"[Title/Abstract] OR lmic[Title/Abstract] OR lmics[Title/Abstract] OR "third world"[Title/Abstract] OR "lami country"[Title/Abstract] OR "lami countries"[Title/Abstract] OR "transitional country"[Title/Abstract] OR "transitional countries"[Title/Abstract] OR "Developing Countries"[Mesh] OR "Rural Population"[Mesh])

**Study Design**

AND (("Double-Blind Method"[Mesh] OR "Single-Blind Method"[Mesh] OR "Cohort Studies"[Mesh] OR "Health Services Research"[Mesh] OR "Time Factors"[Mesh] OR "Clinical Trials as Topic"[Mesh] OR "Controlled Clinical Trials as Topic"[Mesh] OR "Pragmatic Clinical Trials as Topic"[Mesh] OR "Clinical Trial"[Publication Type] OR "Non-Randomized Controlled Trials as Topic"[Mesh] OR "Randomized Controlled Trials as Topic"[Mesh] OR "Pragmatic Clinical Trial"[Publication Type] OR "Comparative Study"[Publication Type] OR "Evaluation Studies"[Publication Type] OR "Evaluation Studies as Topic"[Mesh] OR "Program Evaluation"[Mesh] OR Clinical Trial[Text Word] OR Comparative study[Text Word] OR comparative studies[Text Word] OR Controlled[Text Word] OR evaluation study[Text Word] OR evaluation studies[Text Word] OR follow-up study[Text Word] OR follow-up studies[Text Word] OR longitudinal study[Text Word] OR longitudinal studies[Text Word] OR non-randomised[Text Word] OR non-randomized[Text Word] OR program evaluation[Text Word] OR programme evaluation[Text Word] OR prospective study[Text Word] OR prospective studies[Text Word] OR randomised[Text Word] OR randomized[Text Word] OR quantitative study[Text Word] OR quantitative studies[Text Word] OR quasi experimental[Text Word] OR trial[Text Word] OR trials[Text Word] OR cohort[Text Word])

NOT (animals[mh] NOT humans[mh])

NOT (plants[Text Word] OR Pain[Text Word] OR rats[Text Word] OR mice[Text Word] OR stroke[Text Word] OR qualitative[Text Word] OR United Kingdom[Text Word] OR United States[Text Word] OR America[Text Word] OR American[Text Word] OR Britain[Text Word] OR USA[Text Word] OR British[Title/Abstract] OR New Zealand[Text Word] OR Australia[Text Word])
